# 2018 International Olympic Committee consensus statement on prevention, diagnosis and management of paediatric anterior cruciate ligament (ACL) injuries

**DOI:** 10.1007/s00167-018-4865-y

**Published:** 2018-02-17

**Authors:** Clare L. Ardern, Guri Ekås, Hege Grindem, Håvard Moksnes, Allen Anderson, Franck Chotel, Moises Cohen, Magnus Forssblad, Theodore J. Ganley, Julian A. Feller, Jón Karlsson, Mininder S. Kocher, Robert F. LaPrade, Mike McNamee, Bert Mandelbaum, Lyle Micheli, Nicholas Mohtadi, Bruce Reider, Justin Roe, Romain Seil, Rainer Siebold, Holly J. Silvers-Granelli, Torbjørn Soligard, Erik Witvrouw, Lars Engebretsen

**Affiliations:** 1 Division of Physiotherapy, Linköping University, Linköping, Sweden; 2 School of Allied Health, La Trobe University, Melbourne, Australia; 3 Division of Orthopaedic Surgery, Oslo University Hospital, Oslo, Norway; 4 Oslo Sports Trauma Research Centre (OSTRC), Norwegian School of Sport Sciences, Oslo, Norway; 5 Institute of Clinical Medicine, University of Oslo, Oslo, Norway; 6 Department of Sports Medicine, Norwegian School of Sport Sciences, Oslo, Norway; 7 Department of Pediatric Orthopaedic Surgery, Hôpital Femme Mere Enfant, Lyon, France; 8 Orthopedic Department, Universidade Federal de São Paulo, São Paulo, Brazil; 9 Stockholm Sports Trauma Research Center, Karolinska Institute, Stockholm, Sweden; 10 Department of Orthopaedics, Children’s Hospital of Philadelphia, Philadelphia, USA; 11 OrthoSport Victoria Research Unit, Epworth Healthcare, Melbourne, Australia; 12 College of Science, Health and Engineering, La Trobe University, Melbourne, Australia; 13 Department of Orthopaedics, Sahlgrenska Academy, University of Gothenburg, Gothenburg, Sweden; 14 Division of Sports Medicine, Boston Children’s Hospital, Boston, USA; 15 Harvard Medical School, Boston, USA; 16 Steadman Philippon Research Institute, Vail, USA; 17 The Steadman Clinic, Vail, USA; 18 College of Engineering, Swansea University, Swansea, UK; 19 Santa Monica Orthopaedic and Sports Medicine Group, Los Angeles, USA; 20 The Micheli Center for Sports Injury Prevention, Waltham, USA; 21 University of Calgary Sports Medicine Centre, Calgary, Canada; 22 Department of Orthopaedics and Rehabilitation Medicine, University of Chicago, Chicago, USA; 23 North Sydney Orthopaedic and Sports Medicine Centre, Sydney, Australia; 24 Department of Orthopaedic Surgery, Centre Hospitalier Luxembourg, Luxembourg City, Luxembourg; 25 Sports Medicine Research Laboratory, Luxembourg Institute of Health, Luxembourg City, Luxembourg; 26 Institute for Anatomy and Cell Biology, Ruprecht-Karls-University, Heidelberg, Germany; 27 HKF International Center for Hip, Knee, Foot Surgery and Sportstraumatology, ATOS Klinik, Heidelberg, Germany; 28 FIFA Medical Center of Excellence, Velocity Physical Therapy, Los Angeles, USA; 29 Medical and Scientific Department, International Olympic Committee, Chateau de Vidy, Lausanne, Switzerland; 30 Faculty of Kinesiology, Sports Injury Prevention Centre, University of Calgary, Calgary, Alberta Canada; 31 Department of Rehabilitation Sciences and Physiotherapy, Faculty of Medicine and Healthscience, Ghent University, Ghent, Belgium

**Keywords:** Anterior cruciate ligament, ACL, Orthopaedics, Paediatric, Child, Knee, Consensus

## Abstract

In October 2017, the International Olympic Committee hosted an international expert group of physiotherapists and orthopaedic surgeons who specialise in treating and researching paediatric anterior cruciate ligament (ACL) injuries. Representatives from the American Orthopaedic Society for Sports Medicine, European Paediatric Orthopaedic Society, European Society for Sports Traumatology, Knee Surgery and Arthroscopy, International Society of Arthroscopy Knee Surgery and Orthopaedic Sports Medicine, Pediatric Orthopaedic Society of North America, and Sociedad Latinoamericana de Artroscopia, Rodilla y Deporte attended. Physiotherapists and orthopaedic surgeons with clinical and research experience in the field, and an ethics expert with substantial experience in the area of sports injuries also participated. Injury management is challenging in the current landscape of clinical uncertainty and limited scientific knowledge. Injury management decisions also occur against the backdrop of the complexity of shared decision-making with children and the potential long-term ramifications of the injury. This consensus statement addresses six fundamental clinical questions regarding the prevention, diagnosis, and management of paediatric ACL injuries. The aim of this consensus statement is to provide a comprehensive, evidence-informed summary to support the clinician, and help children with ACL injury and their parents/guardians make the best possible decisions.

**Electronic supplementary material:**

The online version of this article (10.1007/s00167-018-4865-y) contains supplementary material, which is available to authorised users.

## Introduction

The number of anterior cruciate ligament (ACL) injuries in children is rising [112, 133]. ACL injuries in children create a level of concern that is more significant than in any other population with ACL injury. Do children who rupture their ACL mature similarly to their uninjured peers? Do they continue with sport? Do they prioritise their education and other interests over sport? Does an ACL injury and treatment change their lives? These young individuals have to live with their knee problem for the rest of their life, which may compromise their quality of life and increase the risk for further injury, meniscal tears, and early onset osteoarthritis [134]. Compounding the problem is that there is very little high-quality evidence to guide decision-making in management of paediatric ACL injuries [90].

Progress on these issues can only be made based on long-term follow-up in multicentre collaborations. Achieving progress requires a long-term commitment from those who have children’s interests close at heart. Therefore, in October 2017, the International Olympic Committee (IOC) hosted an international expert group of physiotherapists and orthopaedic surgeons who specialise in treating and researching paediatric ACL injuries. Representatives from the following societies attended: American Orthopaedic Society for Sports Medicine (AOSSM), European Paediatric Orthopaedic Society (EPOS), European Society for Sports Traumatology, Knee Surgery and Arthroscopy (ESSKA), International Society of Arthroscopy Knee Surgery and Orthopaedic Sports Medicine (ISAKOS), Pediatric Orthopaedic Society of North America (POSNA) and Sociedad Latinoamericana de Artroscopia, Rodilla y Deporte (SLARD).

Clinicians are charged with the responsibility of providing accurate information and effective treatment to this vulnerable population. Sharing information about the potential consequences of ACL injury and treatment in childhood to long-term knee-health should be a central part of the shared decision-making process. Adult patients with ACL injury may develop symptoms and signs of osteoarthritis within 10 years of the index injury [60]. Therefore, the clinical concern is that a child who is injured at the age of 10 years could have symptomatic osteoarthritis by the age of 20. A quintessential question is what is the long-term prognosis after ACL injury in childhood? Having a definitive, evidence-based answer to this question would strengthen our confidence in clinical decision-making. Clearly the answer to this question is not straightforward and depends on many factors, but one important point is that long-term outcomes after ACL injury in childhood, including the development of osteoarthritis, have not been studied.

Injury management is challenging in the current landscape of clinical uncertainty and limited scientific knowledge. Injury management decisions also occur against the backdrop of the complexity of shared decision-making with children and the potential long-term ramifications of the injury. This consensus statement addresses six fundamental clinical questions regarding the prevention, diagnosis, and management of paediatric ACL injuries (Summary Box 1). By framing each topic around clinical questions, the aim of this consensus statement is to provide a comprehensive, evidence-informed summary to support the clinician, and help children with ACL injury and their parents/guardians make the best possible decisions.

**Summary Box 1:** Six fundamental clinical questions and relevant consensus statement topic(s)

Section 1: How can the clinician prevent ACL injuries in children?
Relevant consensus statement topic: Injury prevention
Section 2: How does the clinician diagnose ACL injuries in children?
Relevant consensus statement topic: Diagnostic tests and imaging
Section 3: What are the treatment options for the child with an ACL injury?
Relevant consensus statement topics: High-quality rehabilitation Surgical techniques The paediatric ACL graft
Section 4: What are the most important considerations when making treatment decisions?
Relevant consensus statement topics: Skeletal age assessment The decision for ACL reconstruction Risks associated with ACL reconstruction Management of associated injuries
Section 5: How does the clinician measure outcomes that are relevant to the child with an ACL injury?
Relevant consensus statement topic: Paediatric patient-reported outcome measures
Section 6: What are the clinician’s role and responsibilities?
Relevant consensus statement topic: Ethical considerations

## Consensus methods

A modified Delphi consensus process [32, 37, 127] was used to identify the topics to be addressed in this consensus statement. Experts were contacted by email in June 2016, and invited to respond to an electronic survey. A mix of open and closed questions was used to gather expert opinion regarding the key issues in the field. These responses were summarised and formed the basis of 18 statements regarding injury prevention, diagnosis, prognosis, surgical techniques, treatment decision-making, management and outcome measurement (Supplementary file 1).

A 2-round consensus process was conducted, involving 19 content experts. Respondents rated the importance of the 18 pre-defined statements on an 11-point scale ranging from *not important at all* to *of utmost importance*. Consensus was defined as a mean ranking of at least 8 points for each statement. After the first voting round, statements reaching consensus were removed, so that only statements that failed to reach consensus in the first voting round went through to the second voting round. The statements that finally reached consensus formed the topics that were discussed at the consensus meeting.

The International Olympic Committee convened a consensus meeting of 21 experts in Lausanne, Switzerland in October 2017. The experts were identified by the IOC through the AOSSM, ESSKA, ISAKOS and SLARD member societies, and from physiotherapists and orthopaedic surgeons with clinical and research experience in the field. An ethics expert with substantial experience in the area of sports injuries also participated.

## Section 1: injury prevention

This section addresses the fundamental clinical question: how can the clinician prevent ACL injuries in children? Prevention of ACL injury is important because of the potential for serious long-term consequences in those who sustain the injury, and because of the increased risk of reinjury to either knee [100]. Therefore, it is paramount that the principles of injury prevention are incorporated in the treatment of the child with ACL injury.

Substantial advances have been made in the development and application of ACL injury prevention programs across numerous pivoting sports. There is compelling evidence that ACL injury prevention programs work in skeletally mature patients—they reduce the number of athletes who sustain a primary ACL injury, and reduce the number of new ACL injuries among athletes who return to sport after primary ACL injury [86, 95, 115, 117, 120, 130].

**Fig. 1:**
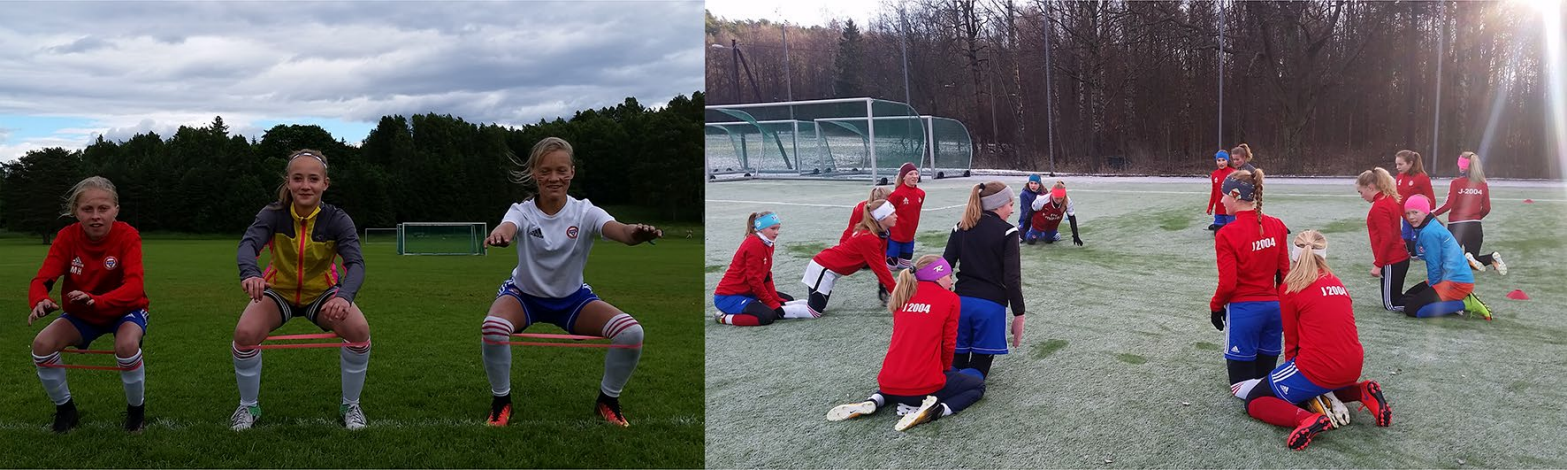
Injury prevention exercises incorporated into team training

The athlete’s biomechanical movement patterns are a key modifiable risk factor for injury. Injury prevention programs target movement patterns by incorporating strength, plyometrics and sports-specific agility training [36, 80]. Coach and athlete education on cutting/landing techniques (e.g., wide foot position when cutting, flexed knee when landing) that avoid high-risk knee positions are also fundamental. Injury prevention programs are straightforward to implement because they require little to no equipment, and are performed as part of regular team training or physical education 2–3 times per week (Fig. 1).

**Fig. 2:**
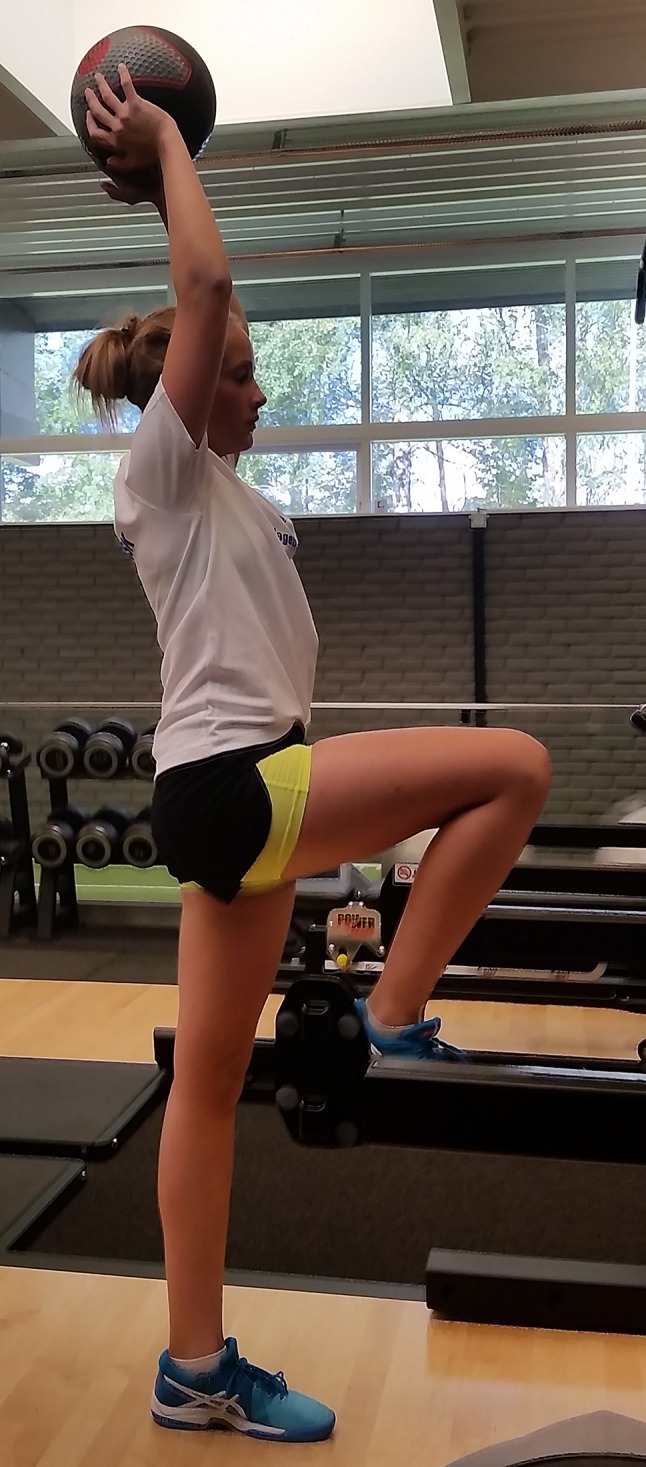
Child demonstrating how to hold terminal knee extension during single limb stance. This is an important marker of quadriceps control in ACL rehabilitation and prehabilitation

### 11+ for kids program

Injury prevention programs should also be implemented early in the athlete’s developmental process. This will give the athlete the best opportunity to develop strong and favourable movement strategies. One well established injury prevention program [126], the 11+, has recently been modified (e.g., adding falling techniques, making partner-based exercises more play-oriented) to suit the paediatric population (FIFA 11+ for kids). Completing the program can reduce football-related lower extremity injuries by over half [107]. Children who complete the program also have improved motor control, balance tests and agility, compared to those who do not complete the program [106].

### Factors that might impact on injury prevention effectiveness

Well-designed injury prevention programs have the lowest injury rates and injury time loss [12, 126]. But the effect of a well-designed injury prevention program is strongly influenced by how frequently athletes perform the training [52, 118, 119]. Therefore, consistent implementation and utilisation, and adherence across all levels of competitive play, is one of the biggest challenges facing the clinician. Those involved in youth sports, and clinicians who treat paediatric athletes with ACL injury have a responsibility to actively advocate for injury prevention in both a primary setting and for children who return to sport after an injury.

## Section 2: diagnosis, clinical tests and imaging

This section addresses the fundamental clinical question: how does the clinician diagnose ACL injury in the child? High-quality injury prevention programs are the first-line defence against the potential negative short- and long-term consequences of ACL injury. However, if injury prevention efforts fail, timely and accurate diagnosis is important, since diagnosis is the starting point for effective management planning and shared decision-making. The clinician combines information from the patient’s history, examination and clinical tests, and imaging to build the clinical picture that will inform diagnosis and treatment. Typically, a thorough history and clinical examination will enable the clinician to make an accurate diagnosis.

*Clinical pearl 1* Haemarthrosis (acute swelling in the knee within 24 h after a trauma due to intra-articular bleeding) following acute knee injury is an important clue suggesting structural knee injury.

*Clinical pearl 2* Diagnosis can be more challenging than in adults because children may be poor historians, they may have greater physiologic joint laxity (be sure to examine both knees), and MRI interpretation is more difficult given developmental variants in children [62, 124].

*Clinical pearl 3* Due to the immature skeleton, children may sustain different knee injuries (e.g., sleeve fracture of the patella, epiphysiolysis) than adults.

Consider starting the assessment by ordering plain knee radiographs for all paediatric patients with a haemarthrosis/suspected acute knee injury. This is because tibial eminence fractures and an ACL tear can present with a similar history and physical examination findings. It is also important to rule out other paediatric fractures (e.g., epiphyseal fracture, sleeve fracture of the patella). Perform an MRI to confirm the diagnosis of ACL injury and evaluate other soft tissue structures [65]. In children with an ACL injury, MRI may yield additional information to identify meniscal tears, other ligament injury, or osteochondral injury. In children with a locked knee, an acute MRI is warranted to assess the presence of a displaced bucket-handle meniscal tear or an osteochondral injury that may need prompt surgical treatment.

### Measurement properties for clinical examination and MRI

No single question, test or image can accurately identify an ACL injury, every time. The measurement tools available to the clinician are not perfect, but they do yield valuable information in the clinical context. Knowledge of the measurement properties of clinical tools helps the clinician balance the information gained from these tools. The negative predictive values of clinical examination and MRI for ACL tear and meniscal pathology are higher than the positive predictive values (Table 1). This means that if the clinical examination or MRI are negative for injury, the chance of the patient having an injury is low. However, if the tests are positive, it does not mean that the clinician can always reliably rule the diagnosis in.

**Table 1:** Diagnostic accuracy of clinical examination and MRI in intraarticular knee disorders (Adapted from Kocher et al. [65])

Diagnosis	Sensitivity (%)			Specificity (%)			Positive predictive value (%)		Negative predictive value (%)	
	Clinical	MRI	*P*	Clinical	MRI	*P*	Clinical	MRI	Clinical	MRI
Anterior cruciate ligament tear	81.3	75.0	0.55	90.6	94.1	0.39	49.0	58.6	97.8	97.1
Medial meniscus tear	62.1	79.3	0.15	80.7	92.0	0.03	14.5	34.3	97.6	98.8
Lateral meniscus tear	50.0	66.7	0.24	89.2	82.8	0.21	34.0	30.1	94.1	95.7

Clinical examination was patient history, physical examination and X-rays performed by a paediatric orthopaedic sports medicine specialist or a post-residency paediatric sports medicine fellow

## Section 3: treatment of ACL injuries in children

This section addresses the fundamental clinical question: what are the treatment options for the child with ACL injury? Once the clinician is certain of the injury diagnosis, he or she first needs to know the available treatment options, and discuss these options with the child and the child’s parents/guardian, so a shared decision can be made about how best to manage the knee injury.

The goals of treatment for the child with ACL injury are:

1. To restore a stable, well-functioning knee that enables a healthy, active lifestyle across the lifespan
2. To reduce the impact of existing or the risk of further meniscal or chondral pathology, degenerative joint changes and the need for future surgical intervention
3. To minimise the risk of growth arrest and femur and tibia deformity

There are two treatment options that can help the child with ACL injury (with or without associated knee injuries) achieve these goals: high-quality rehabilitation alone (non-surgical treatment), and ACL reconstruction plus high-quality rehabilitation. In this section, the key components of high-quality rehabilitation for the child with ACL injury, and the options for ACL reconstruction surgical technique are described. Potential treatment decision modifiers are outlined in “Section 4: treatment decision modifiers”.

### High-quality rehabilitation

High-quality rehabilitation is a critical component in the management of ACL injury, and the principles of rehabilitation are the same, irrespective of whether the child has had an ACL reconstruction or has elected for non-surgical treatment. Guidance for paediatric rehabilitation is extrapolated from clinical experience and research in adults, although it is uncertain whether adult principles apply to children [138]. Paediatric rehabilitation must be performed in close collaboration with the child’s parents/guardians. Exercises and functional goals must be modified, not simply copied from the adult-oriented rehabilitation protocols that may be more familiar to many clinicians. This is because children are not small adults—they cannot be expected to perform unsupervised training independently with perfect technique. Qualified rehabilitation clinicians must supervise rehabilitation for the child with ACL injury.

#### Rehabilitation focus

Dynamic, multi-joint neuromuscular control is the primary focus of ACL rehabilitation in children. For the youngest patients (with markedly open physes, < 12 years), there is less emphasis on the development of muscular strength and hypertrophy. During maturation, and throughout the onset of puberty, rehabilitation strategies that more closely resemble those used with adult patients are appropriate, due to the increase in androgenic hormones [15]. These strategies must include heavier and externally loaded strength training.

Rehabilitation must be thorough, and individualised to the child’s physiological and psychological maturity to achieve successful outcomes. Emphasise exercises that facilitate dynamic lower limb alignment and biomechanically sound movement patterns. Although this has been successfully implemented in the rehabilitation programmes in adolescents and adults, it has not yet been documented as extensively in children. The exercises are gradually progressed through phases 2 and 3 of the paediatric ACL rehabilitation protocol (Summary Box 2; Supplementary file 2) as part of sport-specific rehabilitation. See Supplementary file 2 for examples of exercises to consider in each rehabilitation phase. Reinjury anxiety and the patient’s confidence in his or her injured knee impact on outcomes after ACL rehabilitation in adults [8, 9]. These psychological factors are also likely to be important in the paediatric population, but currently are insufficiently studied.

**Summary Box 2:** Recommended functional tests and return to sport criteria for the child and adolescent with ACL injury

**For patients who choose ACL reconstruction**
Prehabilitation
Full active extension and at least 120° active knee flexion
Little to no effusion
Ability to hold terminal knee extension during single leg standing (Fig. 2)
For adolescents: 90% limb symmetry on muscle strength tests
**For patients who choose ACL reconstruction OR non-surgical treatment**
*Phase 1 to phase 2*
Full active knee extension and 120° active knee flexion
Little to no effusion
Ability to hold terminal knee extension during single leg standing
*Phase 2 to phase 3*
Full knee range of motion
80% limb symmetry on single leg hop tests, with adequate landing strategies
Ability to jog for 10 min with good form and no subsequent effusion
**For adolescents**: 80% limb symmetry on muscle strength tests
*Phase 3 to 4: sport participation (return to sport criteria), and continued injury prevention*
Single-leg hop tests > 90% of the contralateral limb (with adequate strategy and movement quality)
Performed gradual increase in sport-specific training without pain and effusion
Confident in knee function
Knowledge of high injury-risk knee positioning, and ability to maintain low-risk knee positioning in advanced sport-specific actions
Mentally ready to return to sport
**For adolescents**: 90% limb symmetry on muscle strength tests

Muscle strength testing should be performed using isokinetic dynamometry or handheld dynamometry/1 repetition maximum. The type of test and experience of the tester are highly likely to influence the results. If using handheld dynamometry/1 repetition maximum, consider increasing the limb symmetry criterion cut-off by 10% (i.e. 90% limb symmetry becomes 100% limb symmetry). Clinicians who do not have access to appropriate strength assessment equipment should consider referring the patient elsewhere for strength evaluation

Following surgical treatment, the graft type used for ACL reconstruction, and associated injury or surgery to other ligaments, menisci or articular cartilage, necessitate specific adjustments to the rehabilitation program. Rehabilitation programs should be designed to allow the child to participate in his or her team training sessions to maintain the social benefits of staying within the team. Parents or guardians should be active participants in the daily rehabilitation [101]. This may include assisting the child in technical and functional exercises during team training (e.g., short passes in football/soccer).

#### Rehabilitation phases

Rehabilitation for the child with an ACL injury is organised into four phases (Summary Box 2, Supplementary file 2), with an additional prehabilitation phase for those who choose ACL reconstruction. Specific clinical and functional milestones should be met before progressing from one phase to the next [128]. Throughout the first two phases, the child should be guarded from cutting and pivoting activities during sport, free play and physical education classes in school.

#### Rehabilitation progression

The framework for progression through functional milestones is similar for ACL reconstruction and non-surgical treatment. However, there are different expectations for progression and time to return to full participation in sport. For all patients, rehabilitation progression must be guided by clinical and functional milestones (Summary Box 2), and return to full participation [7] is dependent on successfully achieving the return to sport criteria (Summary Box 2). Non-surgical treatment should last for at least 3–6 months [49]. Post-operative rehabilitation should last for a minimum of 9 months before return to full participation in preferred physical activities [50].

Data from international registries suggest that young athletes are at high-risk for a second ACL injury following an ACL reconstruction [76], and the risk is highest in the first 12 post-operative months [28, 50]. Therefore, consider advising the child athlete not to return to pivoting sport until at least 12 months following ACL reconstruction. Rehabilitation is also an excellent opportunity to train the uninjured leg, which might be important considering the risk of contralateral injury [28]. Once the child returns to sport, a comprehensive injury prevention program, emphasising biomechanical alignment and landing/cutting technique should be integrated with usual training.

#### Five considerations when designing rehabilitation programs for the prepubescent child

**Fig. 3:**
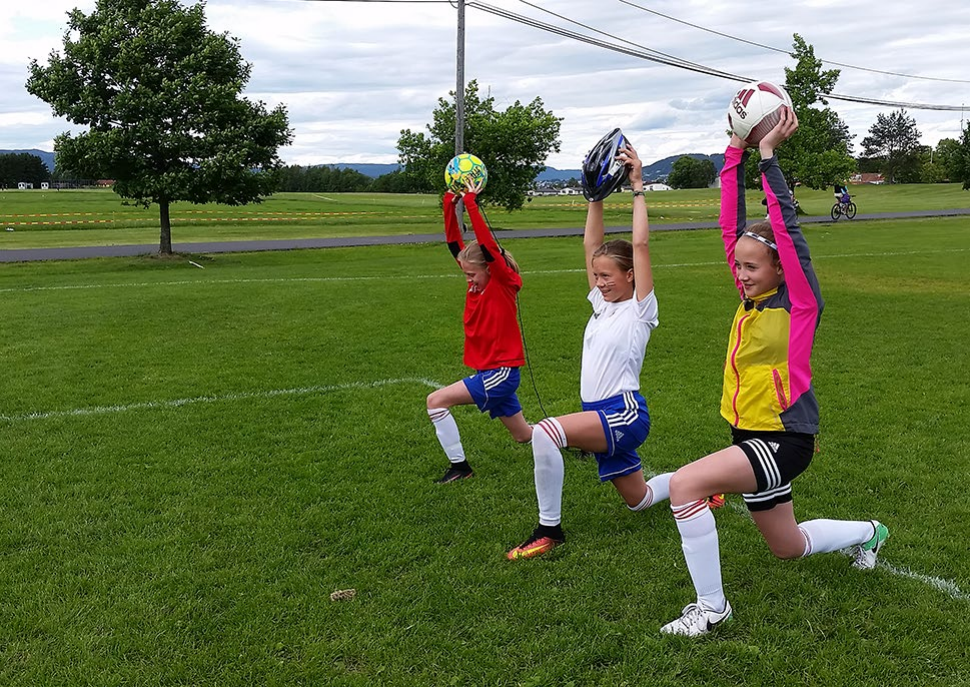
One example of an exercise that could be incorporated into a home-based ACL rehabilitation program

Children who are close to skeletal maturity may follow rehabilitation [128] and RTS guidelines [50, 77] intended for adults. There are five important considerations for the prepubescent child:

1. Consider a home-based program, with emphasis on playful exercises and variation (Fig. 3) to discourage boredom.
2. Single leg hop tests and isokinetic strength tests have larger measurement errors in the prepubescent population, so use these tests with caution [59].
3. Focus on evaluating the quality of movements during single leg hop testing, instead of the leg symmetry index (LSI) measures.
4. Tests and criteria to assess movement quality are yet to be validated, so the responsible clinician needs to have skills and experience in this area.
5. Return to sport criteria were designed and scientifically tested in the skeletally mature patient and are recommended for the child who is close to maturity [50, 125]. The validity of these criteria in the prepubescent child is unknown.

#### Bracing

Many clinicians involved in non-surgical treatment of skeletally immature children recommend the child wear a protective brace during strenuous physical activities [92]. The child who has had surgical treatment typically wears a brace during the prehabilitation phase, until ACL reconstruction is performed. Following surgery, it is recommended that the child wears a protective knee brace through the successful completion of the functional milestones in rehabilitation phase 1 (usually 2–6 weeks post-operative, depending on concomitant surgical procedures). However, the effectiveness of bracing following ACL injuries or reconstruction in paediatric patients is unknown. Other considerations related to the use of a brace might be to prevent knee hyperextension or knee valgus/varus, to enhance the child’s awareness of his or her injury, and as a protective signal to others the child might encounter (e.g., at school).

### Surgical techniques

The general principles of ACL reconstruction in adults also apply to the paediatric patient: use a well-positioned (soft tissue) autograft of adequate size, with adequate fixation to allow functional rehabilitation. Physeal damage should be minimised to avoid growth disturbance. Bone plugs and fixation devices should not cross the physis [41, 68, 111].

#### Key indications for ACL reconstruction

There are three indications for paediatric ACL reconstruction:

1. The child has repairable associated injuries that require surgery (e.g., bucket-handle meniscus tear, repairable meniscal lesion or osteochondral defect)
2. The child has recurrent, symptomatic knee giving way after completing high-quality rehabilitation
3. The child experiences unacceptable participation restrictions (i.e., an unacceptable modification of activity level to avoid knee giving way)

There are three possible techniques for paediatric ACL reconstruction:

### Transphyseal ACL reconstruction

**Fig. 4:**
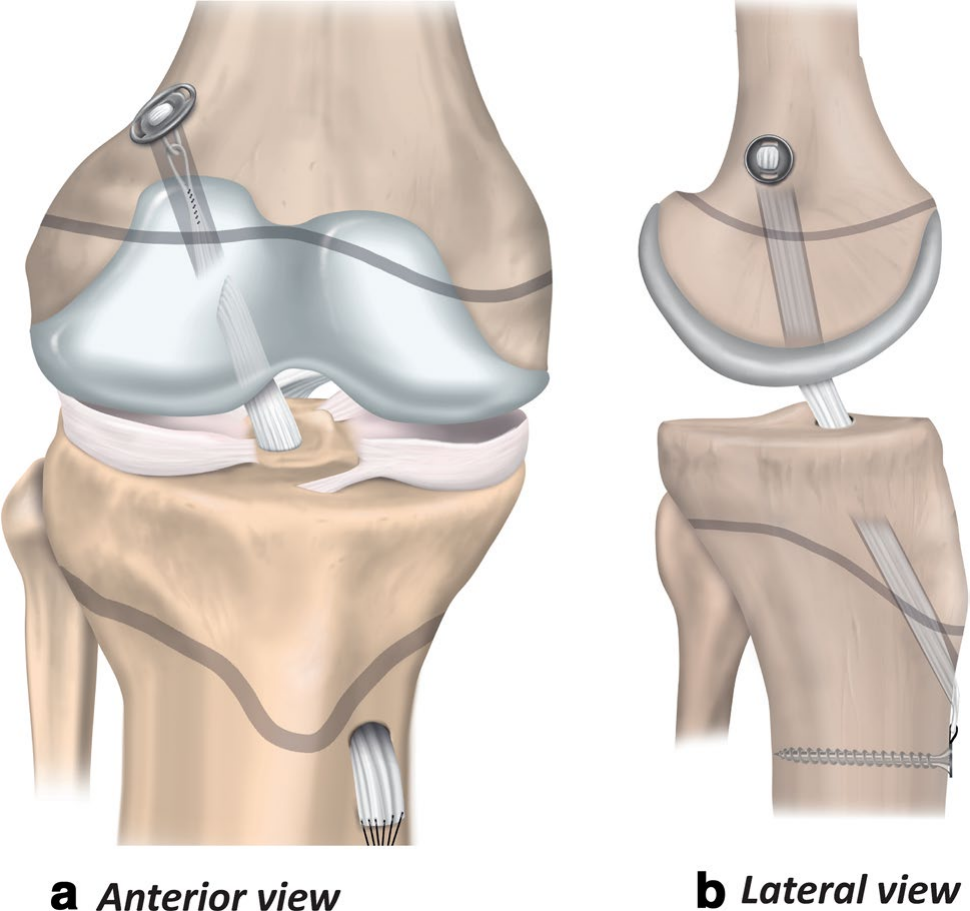
Transphyseal ACL reconstruction (anterior and lateral views)

The transphyseal technique in the child is similar to the technique the surgeon would use for ACL reconstruction with adults. Single bundle transphyseal ACL reconstruction with a quadrupled hamstring graft is the most common (Fig. 4) [21, 25, 38, 55, 71, 114]. Therefore, because the surgeon is more likely to be familiar with the key elements of the procedure, it may reduce the risk of intraoperative complications. Ensure the diameter of the bone tunnels is as small as possible (< 9 mm) to accommodate an appropriate size graft [58]. Similarly, to minimise physeal damage, orient the tibial tunnel as vertically and as centrally as possible while maintaining the anatomical position of the graft. On the femoral side, the surgeon should take care to avoid the perichondral ring. Drilling via the anteromedial portal can result in a tunnel that has an elliptical trajectory through the physis. Consider a slightly more vertical orientation than might be used for an ACL reconstruction in an adult patient, or choose a different drilling approach.

### Physeal-sparing ACL reconstruction

**Fig. 5:**
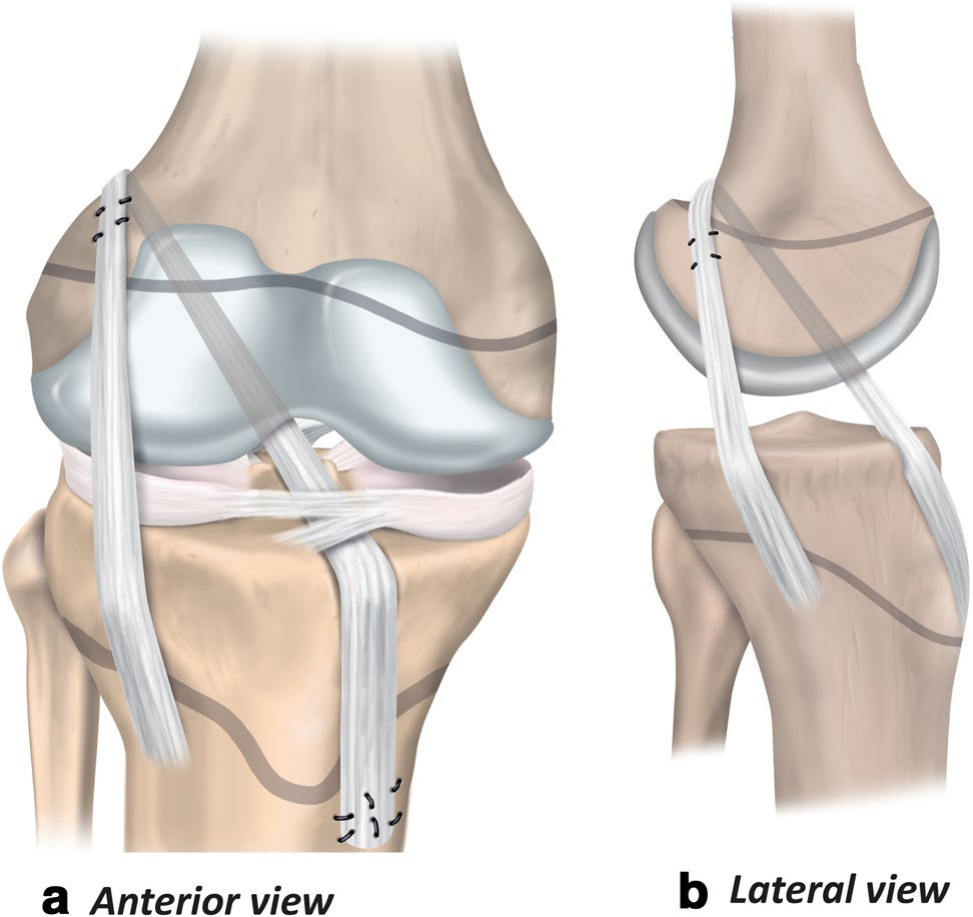
Physeal-sparing ACL reconstruction using an over-the-top technique with iliotibial band (anterior and lateral views)

**Fig. 6:**
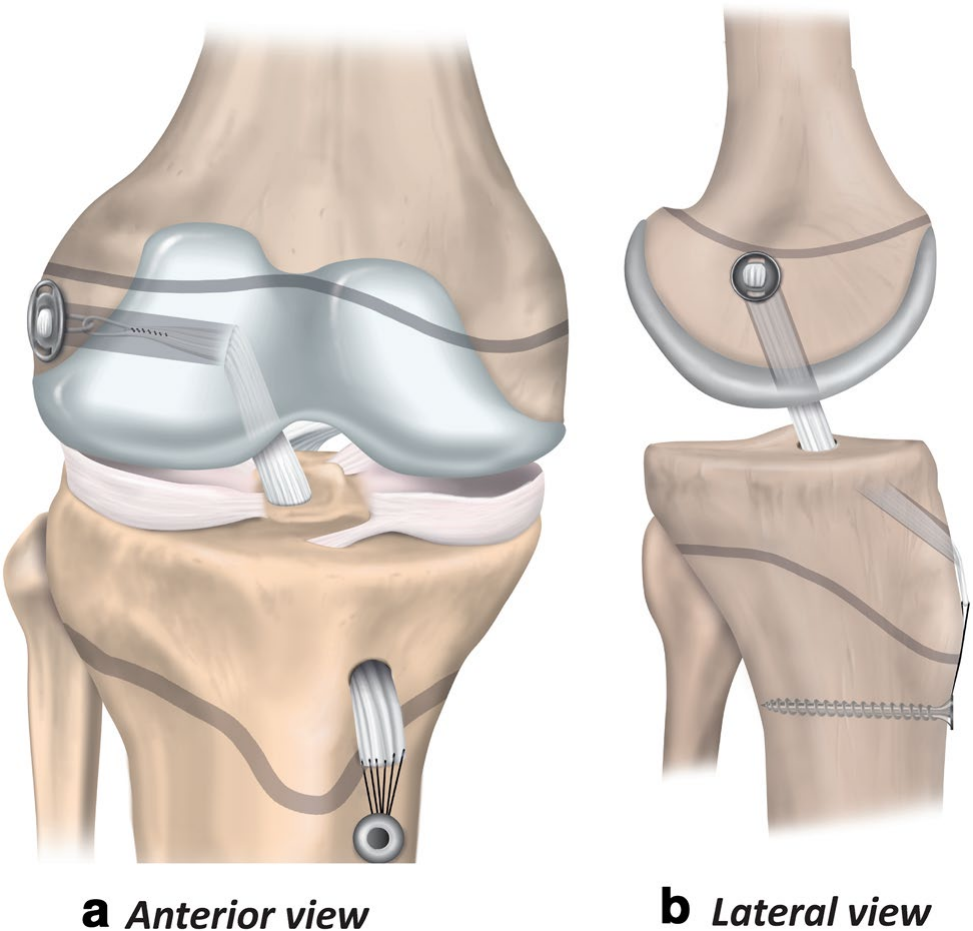
Physeal-sparing ACL reconstruction using an all-epiphyseal technique (anterior and lateral views)

Physeal-sparing techniques avoid physeal damage in patients with markedly open physes. The techniques include an over-the-top technique with a strip of the iliotibial band (Fig. 5) [67], and an all-epiphyseal procedure (Fig. 6) [3]. In the all-epiphyseal procedures, use of fluoroscopic visualisation is recommended to reduce the risk of physeal damage. When using the over-the-top technique, avoid femoral rasping to minimise the risk for damage to the perichondral ring.

### Partial transphyseal ACL reconstruction

**Fig. 7:**
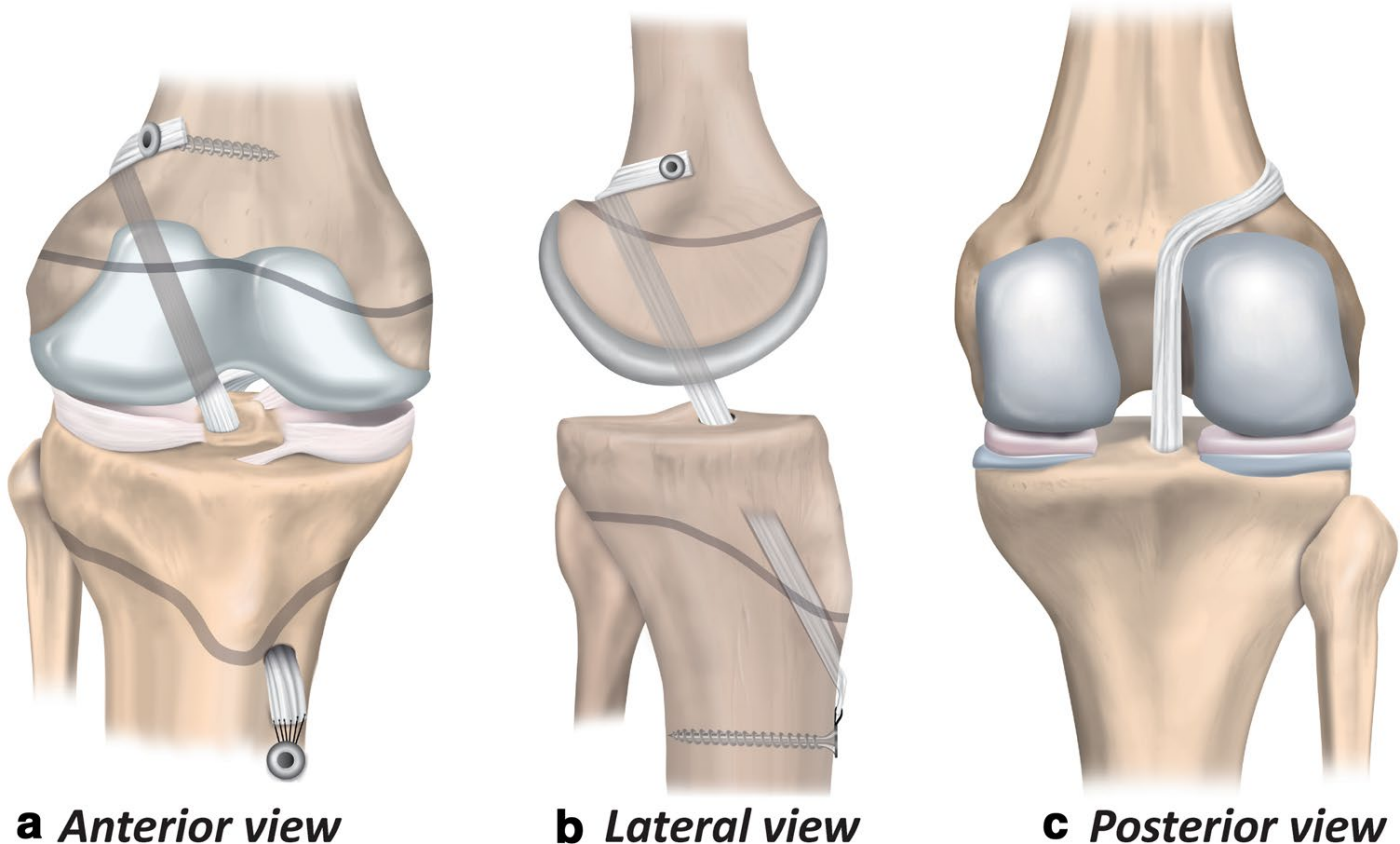
Partial transphyseal ACL reconstruction (anterior, lateral and posterior views)

The partial transphyseal technique (Fig. 7) combines a transphyseal tibial tunnel with a physeal-sparing technique on the femoral side [5, 53, 82].

### Surgical principles and techniques for growth disturbance risk reduction

**Fig. 8:**
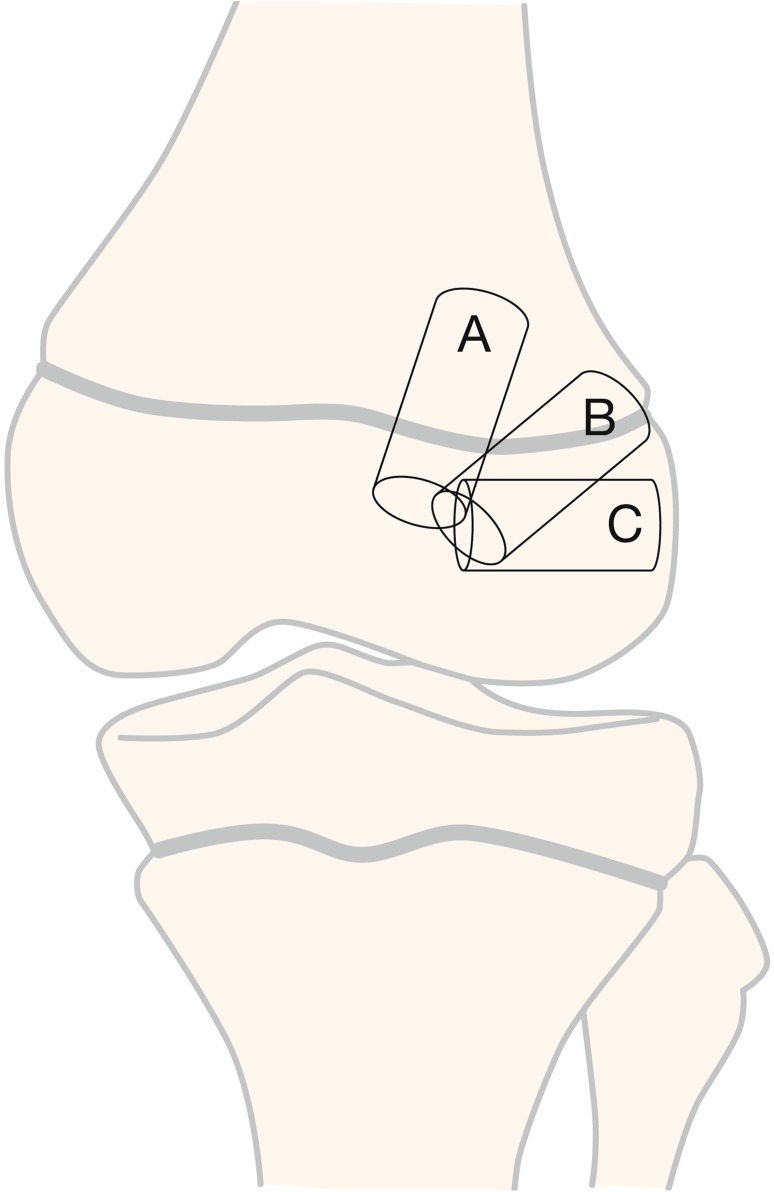
Three options for femoral tunnel trajectories

Drill hole trajectory and location influence the degree of risk to the physes (Summary Box 3; Fig. 8). Knowledge of three key principles will help the surgeon minimise the risk to the physes during transphyseal ACL reconstruction:

**Summary Box 3:** Three different options for femoral tunnel trajectory

Tunnel option A: vertical transphyseal
Advantage: minimises physeal volume affected Disadvantage: less than ideal coverage of ACL footprint
Tunnel option B: oblique transphyseal
Advantage: anatomical graft position covering the ACL footprint Disadvantage: greater volume of physis negatively affected
Tunnel option C: horizontal all-epiphyseal
Advantage: appropriate placement at ACL footprint; no drilling through the physis Disadvantage: requires precise tunnel placement to reduce the risk for physeal damage

1. Drilling at the periphery of the physis and the perichondral ring increases the risk of growth disturbance. Drill holes may be placed in an all-epiphyseal manner to allow for drilling at the native ACL footprint, while avoiding the physis. Precise tunnel placement is required when performing this technique to avoid damage to the undulating distal femoral physis.
2. Bone tunnel drill holes should be as vertical as possible (while still maintaining anatomic graft position) and as central as possible. This is especially important when drilling through the anteromedial portal. Drilling an oblique tunnel rather than a more vertical tunnel increases the amount of physis removed and increases the risk for growth disturbance.
3. Do not cross the epiphysis with hardware, implants or bone blocks. Fill bone tunnels with soft tissue, rather than leaving the tunnels open.

#### Graft choice and fixation

Only soft tissue grafts (not allografts) should be used for ACL reconstruction in paediatric patients with open physes. The quadrupled hamstring graft is most common [25, 38, 55, 71, 114]. A quadriceps tendon graft may be used [53]. The patella tendon should not be harvested in paediatric patients with open growth plates to avoid damage to the tibial tubercle apophysis. Allografts are not indicated in paediatric patients in most cases, since the use of allografts in paediatric ACL reconstruction has poor clinical outcomes [61, 108, 123]. A novel technique involving the use of living-donor hamstring tendon allograft has been reported [47, 55] to avoid the varied sterilisation techniques used in cadaveric soft-tissue allografts, and preserve of the neuromuscular unit of the growing patient [139, 140]. However, long-term clinical outcomes are yet to be assessed.

Extracortical fixation of soft tissue grafts may be performed with a cortical button, suture, post or staple. Aperture fixation may be performed with interference screws, provided the screws do not cross the physis.

#### Graft incorporation

Data regarding ACL graft incorporation in children are scarce. Paediatric soft tissues have a greater biological growth potential compared to adults [40, 94], and cell migration and proliferation of ACL-fibroblasts slows as the person grows older [83]. The clinical relevance of the growth potential to paediatric ACL reconstruction is still unclear [102], although there is a rationale from animal models that the paediatric ACL graft may remodel faster than the adult ACL graft [89].

#### Adaptations and remodelling in the growing child

The ACL graft must adapt as the child grows. The graft may increase in length as the bone grows, and the bone tunnels may reduce in relative size [16, 73]. It is uncertain whether the diameter of the intraarticular part of the graft becomes longer and thinner [11], or not [16], as the child grows. The graft does not increase diameter as the child grows, but may increase in length [10].

With longitudinal bone growth after transphyseal ACL reconstruction, the graft may become more vertically oriented. This observation might be explained by the movement of the femoral fixation site with physeal growth or because the tibial tunnel aperture becomes relatively more posterior due to greater anterior growth of the proximal tibia. Other changes occurring as the child grows are secondary intercondylar notch narrowing, distal migration of the tibial and/or proximal migration of the femoral extracortical fixations, and verticalisation of the Blumensaat line [110]. However, the long-term clinical significance of these growth-related changes is unclear.

## Section 4: treatment decision modifiers

This section addresses the fundamental clinical question: what are the most important considerations when making treatment decisions? The key issues addressed relate to assessment of skeletal maturity, the decision for surgery or not, management of injuries to other knee structures and potential adverse events following treatment. These issues may alter the ACL injury management decision, depending on the decision-making team’s [which should include clinicians, the child and the child’s parent/guardian(s)] risk tolerance.

### Skeletal age assessment

Assessing and documenting the child’s skeletal age, in addition to his or her chronological age, is necessary to individualise treatment of ACL injuries. The main goal with respect to skeletal age assessment is to define remaining knee growth. Protecting the physis and perichondral ring from damage during ACL reconstruction is an important consideration [111]—an insult to a growth area that is near completion of growth can result in premature closure.

Estimating skeletal age and remaining growth are key considerations for treatment decision-making. These estimates will guide choice of treatment, timing of surgery and surgical method. Open physes in the child are vulnerable at surgery, and none of the current recommended surgical treatments for the child with an ACL injury can be guaranteed to protect the physis and avoid the potential complication of growth arrest or deformity (these risks are outlined below). The clinician might also consider long leg radiographs (hips to ankles) after injury to establish a baseline for assessing the potential development of angular deformity and leg length discrepancy. Assessing skeletal age is also relevant in research and may be beneficial for medicolegal reasons. If overgrowth, growth arrest or deformity occurs, pre-surgical documentation of skeletal age may be important.

Five considerations for skeletal age assessment:

1. Understand the difference between skeletal age and chronological age.
2. Use imaging of the knee to determine if the femoral and tibial physes, and the tibial tubercle apophysis are open. If the growth areas are closed, then, independent of chronological age, the child can be treated as an adult.
3. None of the specific methods for skeletal age determination in isolation are sufficient to accurately determine skeletal age.
4. Use a multifaceted clinical approach to determining skeletal age that includes whether or not the child has had an adolescent growth spurt, the relative heights of the child’s parents and Tanner staging [122].
5. The most common method of skeletal age assessment is via posterior-anterior left hand and wrist X-ray. This can be compared to a skeletal atlas (e.g., Gilsanz and Ratib [46] or Greulich and Pyle [48]) or using a smart-phone application (e.g., the Bone Age app for iPhone).

### Treating the child with ACL injury: to operate or not to operate?

Children who have repairable additional injuries at ACL injury diagnosis (e.g., displaced bucket-handle meniscal tear) should be treated with early ACL reconstruction and meniscal repair [75]. In those without additional injuries warranting surgery, there are conflicting opinions regarding the best treatment approach. These approaches range from early ACL reconstruction for all children, to primary non-surgical management (high-quality rehabilitation alone) with the option of late ACL reconstruction if the child has recurrent instability problems despite high-quality rehabilitation or if he or she sustains secondary intraarticular injuries.

A well-performed ACL reconstruction and preservation of the meniscus can restore knee stability [66]. However, if the child receives inadequate (or no) rehabilitation, the chances of recovering high-level function to safely participate in all aspects of life (including pivoting sports), for the rest of his or her life, might be slim. Similarly, high-quality rehabilitation will not salvage poor surgical treatment (e.g., graft malposition).

Children who undergo ACL reconstruction after failed non-surgical management may have a higher number of meniscal and chondral injuries at the time of ACL reconstruction compared with those who undergo early ACL reconstruction [4, 81, 97]. The number of instability episodes prior to surgery appears to be a more important factor than the length of time between injury and surgery [42]. This consideration is the background for early surgery decisions. However, there are a lack of high-quality, prospective studies investigating the outcomes of surgical and non-surgical treatment for paediatric ACL tears [90].

Non-surgical treatment is a viable and safe treatment option in skeletally immature patients who do not have associated injuries or major instability problems [93]. High-quality rehabilitation alone may stabilise the knee dynamically without compromising the physes, and is a focused training programme supervised by a qualified rehabilitation clinician (see “Section 3: treatment of ACL injuries in children” for the key principles of high-quality rehabilitation). Non-surgical treatment can be a permanent treatment option for those who do not develop functional instability, or a short-term option to delay ACL reconstruction until the child has reached skeletal maturity. Abandoning non-surgical treatment in favour of ACL reconstruction is an option if the child has recurrent instability problems despite completing high-quality rehabilitation, or if the child has a secondary intraarticular injury. Therefore, clinicians must work together to closely and frequently monitor the child with repeated MRI and clinical examination as appropriate, being alert to instability episodes and secondary injuries that require prompt assessment and treatment [42].

### Risks associated with ACL reconstruction

Irrespective of the technique, surgical treatment of the ACL has inherent risks. Different ACL reconstruction techniques have different considerations to help avoid risk to the physes, articular surface and soft tissue structures of the knee. Here we describe five key risks associated with surgical treatment for ACL injury of which clinicians, patients and their parents/guardians must be aware.

#### Risk 1: growth disturbance

Growth disturbances are a rare (approximately 2%) [41], but serious risk of ACL reconstruction. Growth disturbances may be a result of hardware, bone plugs at the physis, extra-articular tenodesis, or use of over-the-top femoral position. Most of the growth in the child’s lower extremities occurs from the physes of the distal femur and proximal tibia. Any surgical procedures, where tunnels are drilled through or near the physis are associated with a risk of growth arrest, and associated angular deformity and/or leg length discrepancy. Transphyseal techniques have a higher rate of graft rupture and a lower rate of lower limb deformity or axis deviation. Physeal-sparing techniques have a lower rate of graft rupture, and a higher rate of lower limb deformity or axis deviation.

Highly tensioned soft tissue grafts placed across femoral physes have been associated with limb length discrepancy and angular deformity [34]. Metaphyseal fixation techniques may pose an increased risk of femoral angulation and rotation relative to other techniques. Epiphyseal techniques may increase the risk of rotational deformity and decrease the risk of angular deformity [23]. Excessive growth may also be a problem, including symmetrical and asymmetrical overgrowth [22].

Most patients with ACL rupture requiring surgical treatment are approaching skeletal maturity, and do not have substantial growth remaining. This means that angular deformities and limb length discrepancies are likely of relatively low clinical significance. Therefore, it may be reasonable to perform transphyseal procedures when the child has minimal growth remaining.

#### Regularly monitor the patient until skeletal maturity

Routine clinical and radiological follow-up within the first 12 post-operative months can help the surgeon detect early clinical and radiographic evidence of leg length discrepancy, angular deformity or and physeal injury. For the child with markedly open physes, appropriate follow-up evaluation of leg length discrepancy might include annual clinical assessment and knee radiographs with long-leg alignment views until skeletal maturity and physeal closure. Height should be monitored, and if growth exceeds 6 cm in 6 months, or if clinical findings warrant, the annual assessment should be brought forward.

#### Classifying growth disturbances

**Fig. 9:**
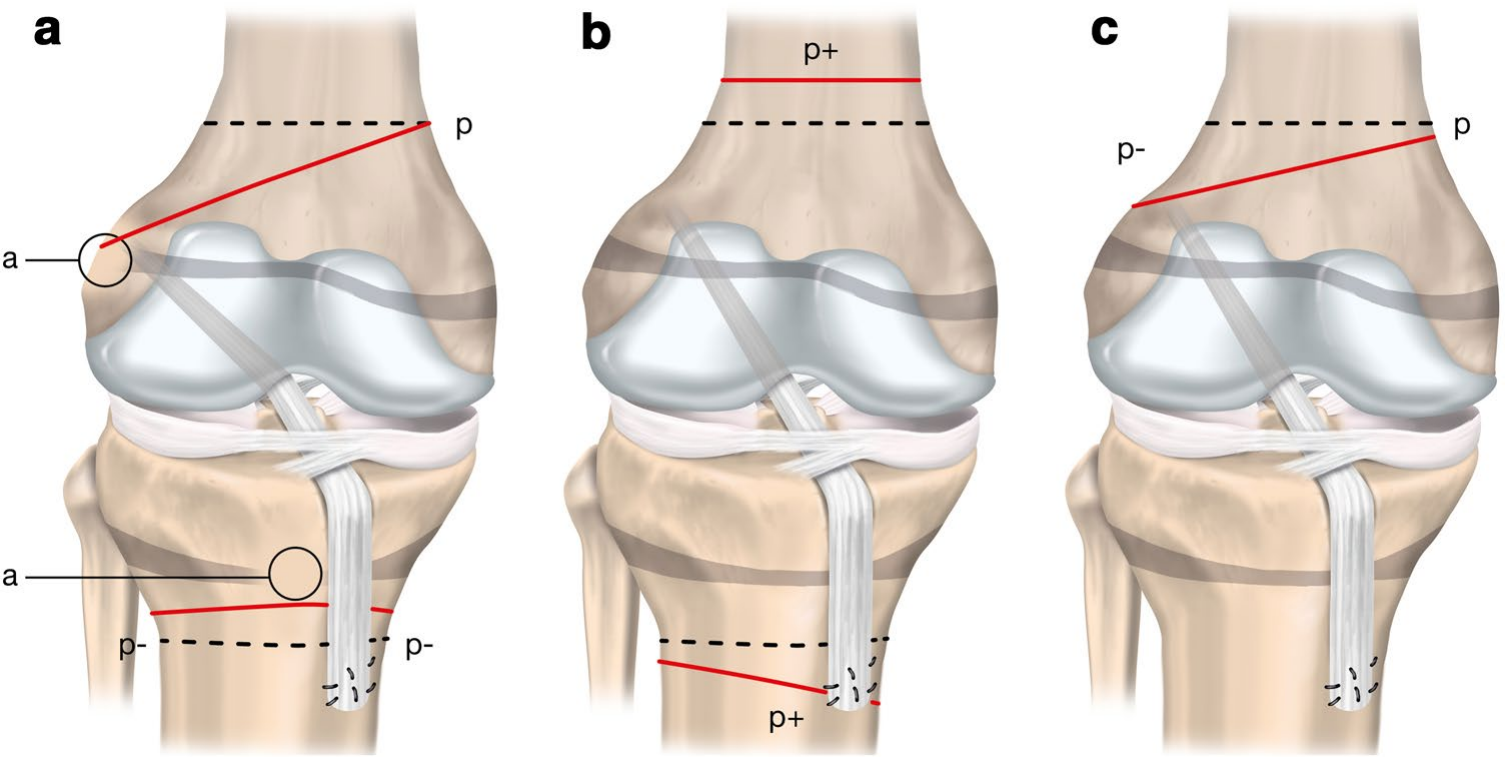
Three growth disturbances that may occur following ACL reconstruction. “p” represents the physiological growth process; dashed lines represent the physiological growth arrest lines; continuous lines represent the observed pathological growth arrest line. Type A (Arrest): growth arrest process (a) occurs after a localised injury of the physis and results in a bone bridge across the physis. The extent of deformity is proportional to the location and size of the initial physeal injury. Type B (Boost): overgrowth process (p+) is probably caused by local hypervascularisation, stimulating the open physis (b). This growth disturbance is temporary and usually becomes apparent in a limited period of 2 years following ACL reconstruction. It primarily leads to leg length discrepancy. Type C (deCelerate): undergrowth process (indicated by p−) due to a tenoepiphysiodesis effect. The graft tension across the open physis causes the deformity. Adapted from [22]

Growth disturbances can occur in several different forms (Fig. 9). The growth arrest may be due to:

- Localised physis injury resulting in a bone bridge leading to growth arrest and possible malalignment (Type A).
- Overgrowth process potentially caused by hypervascularisation (Type B).
- Undergrowth process arising from a graft traversing a physis under tension during growth and leading to a tethering effect (Type C).

#### Risk 2: secondary ACL rupture

Young age, returning to pivoting sport and receiving an allograft are important predictors of new ACL injury after index ACL reconstruction [2, 61]. One in four patients under 25 years who returned to pivoting sports after ACL reconstruction can be expected to sustain a new ACL injury (the pooled ipsilateral reinjury rate is approximately 10%; the pooled contralateral reinjury rate is approximately 12%) [135].

High rates of reinjury among young people with ACL reconstruction are concerning, although data regarding reinjuries among children with ACL reconstruction are sparse in comparison to data from skeletally mature patients. The best available evidence suggests a graft rupture rate in children and adolescents (age range 6–19 years) of 13%, and a contralateral ACL injury rate of 14% [63]. It is reasonable to hypothesise that high-quality rehabilitation with high adherence is likely an important step in reducing reinjury risk. The principles of rehabilitation for the skeletally immature patient are addressed in “Section 3: treatment of ACL injuries in children”. The ACL graft is also affected by the status of the other ligaments, menisci, cartilage surfaces, limb alignment, rotation and the dynamic muscle control of these structures—all factors that must be considered during treatment decision-making.

#### Risk 3: poor long-term knee-health

Meniscectomy is associated with an increased risk for osteoarthritis [24, 103, 137]. Therefore, wherever possible, treatment of ACL injuries must emphasise preservation of the meniscus. Prior meniscectomy at the time of ACL reconstruction is associated with higher likelihood of chondral lesions, while prior meniscal repair is not associated with a higher likelihood of chondral lesions [19]. Because of the technical nature of performing ACL and concurrent meniscal surgery in smaller, younger patients with open physes, patients in whom meniscus repair is indicated should be treated by surgeons who (1) are experienced in treating patients with open physes, and (2) perform a high volume of meniscal repairs.

#### Risk 4: knee stiffness

Knee stiffness may be due to the degree of injury to the ACL, disruption of the joint capsule, and injury to structures other than the ACL. Knee stiffness may also be related to surgical interventions or inadequate rehabilitation. Knee stiffness is rare in children aged 13 years and younger, and less common in males and in those having surgery with an iliotibial band or hamstring autograft [98]. Patients who have knee stiffness following ACL injury should aim for full active knee extension range of motion prior to undergoing ACL reconstruction. If the knee extension deficit persists beyond 3 months post-operative, MRI to assess for anterior impingement (cyclops lesion), and subsequent arthroscopy (should the deficit continue to be unresolved despite focused rehabilitation attention) may be warranted.

#### Risk 5: infection

Data related to infection risks for paediatric patients are extrapolated from literature that combines paediatric and adult patients. Infection rates in adult patients are generally low for ACL reconstruction. The rate of deep infections after ACL reconstruction with autograft is 0.19% [13].

### Management of associated injuries

In this section, the key issues for managing cartilage and meniscal injuries in combination with ACL rupture, and the multiligament-injured knee are addressed.

#### Associated meniscus and cartilage injuries in children with ACL injuries

**Fig. 10:**
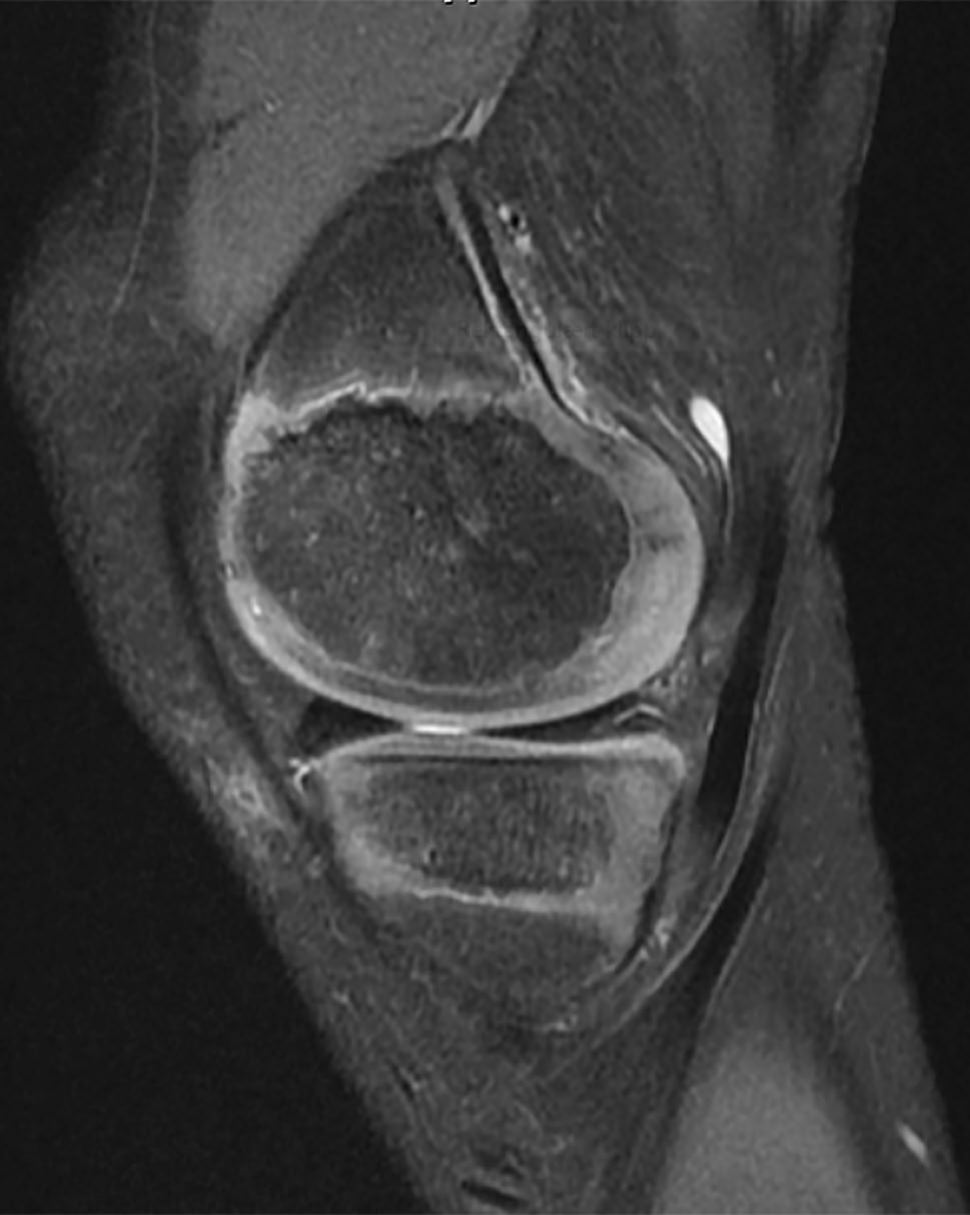
Appearance of the highly vascular paediatric meniscus on MRI. 10-year-old boy, 3.0T MRI (Signa HDxt 3.0-T; GE Medical Systems)

The degree of vascular penetration of the menisci declines with age to between 10 and 30% of the menisci receiving vascular inflow in adults [104]. The more robust vascular distribution in the paediatric menisci is reflected by increased intrameniscal signal intensity on MRI. Globular and intrameniscal signal may be observed in children and may appear to be an intrasubstance meniscal tear. However, these findings are benign, and usually reflect the abundant vascularity of the paediatric menisci (Fig. 10) [26].

It is important to evaluate the MRI characteristics of the paediatric menisci to rule out meniscal injuries. In cases where the diagnosis is difficult, a diagnostic arthroscopy may be performed to clarify the diagnosis and ascertain the state of the meniscus. The clinician should also assess for a posterior medial meniscocapsular tear (ramp lesion). Ramp lesions may be present in 1 in 6 adult patients with ACL injury, and the prevalence of ramp lesions in children with an ACL injury is similar to adults [84]. The surgeon should be vigilant to verify the presence or absence of a medial meniscal ramp tear by visualising the posteromedial compartment. Use a posteromedial knee arthroscopic portal, if necessary, to probe the posteromedial meniscocapsular junction. Ramp lesions may place more stress on an ACL reconstruction if the lesion is not concurrently repaired [30].

Meniscal repair should be performed whenever possible in the paediatric patient because of the deleterious effects of meniscectomy and the positive outcomes of meniscal repair (i.e., the improved healing potential of the meniscus) [4, 74, 113]. This is especially important for bucket-handle, root and radial meniscal tears, and ramp lesions. If the surgeon does not have the skills or equipment to repair the meniscus tear, he or she should consider referring to a surgeon who has the expertise and equipment. Early diagnosis and appropriate treatment of ACL injuries and meniscus tears is needed to provide the best chance of preserving meniscal tissue.

Articular cartilage injuries in combination with ACL injury are less common than meniscal tears [4]. However, the clinician should have a higher degree of suspicion of articular cartilage injury in patients with combined ACL and meniscal injuries [33]. The medial femoral condyle may be particularly vulnerable [33]. Factors that may be associated with more severe chondral lesions are recurrent instability episodes and increased time between ACL injury and reconstruction [33, 51, 81]. It is unclear whether non-surgical management of ACL injuries is associated with a higher incidence of new chondral and meniscal lesions than ACL reconstruction [91].

#### Associated ligament injuries in children with ACL injuries

There is limited research on multiligament knee injuries and treatment in paediatric patients, and these injuries are less common in children than in adults [87]. Therefore, consider referral to a specialist centre.

#### Specific surgical treatment considerations

*Combined ACL and fibular collateral ligament injuries* Use fluoroscopy prior to placing suture anchors for a repair, or for tunnel reaming for a concurrent ligament reconstruction, to evaluate tunnel position in relation to the physes [136].

*Combined ACL and posterior cruciate ligament (PCL) injuries* Non-surgical treatment may be appropriate for partial PCL tears or non-displaced avulsion injuries. PCL reconstruction is a relatively safe and viable treatment option in patients with multiligament injuries [69]. Using a tibial inlay technique with a modified femoral tunnel location avoids transphyseal drilling [132]. Although, there are no high-quality studies of this technique in children.

*True knee dislocation* Perform a reduction by manipulating the tibia relative to the femur. Avoid forceful hypertension or rotation, to minimise the risk for damage to cartilaginous and/or neurovascular structures. Following reduction, a dynamic knee brace can be applied (for at least 12 weeks) to prevent further intraarticular damage and to help hold the knee reduced [79] while further treatment is planned. Ultimately, reconstruction of the ACL and PCL in combination with repair/reconstruction of additional ligaments (as needed) is the appropriate treatment.

## Section 5: paediatric patient-reported outcome measures (PROMs)

This section addresses the fundamental clinical question: how does the clinician measure outcomes that are relevant to the child with ACL injury? Assessing PROMs provides insights into aspects of the patient’s function that cannot be evaluated with clinical tests or imaging [27]. Because of this, evaluating PROMs is important when managing the child with an ACL injury, and when conducting research in this field.

Valid outcome instruments must have appropriate measurement properties, including reliability, validity (content, criterion, and construct), and responsiveness. Instruments that were developed for adults may not be valid for children and adolescents. Paediatric patients have different levels of comprehension (this age group includes a spectrum of comprehension abilities from younger children to older adolescents), and interpretation of instruments. Most importantly, paediatric patients may value different outcomes when evaluating their knee function, and instruments must reflect the issues that are important to children and adolescents.

Paediatric PROMs should be either developed, or specifically validated in this population. The process of validation should include an assessment of comprehensibility, reliability, validity, and responsiveness. Child-reported outcome assessment is typically valid in older children and adolescents (≥ 10 years) [116]. In younger children (< 10 years), parent-proxy-reported outcome assessment may be more appropriate. However, there is potential for bias with proxy-reported outcomes [109].

Paediatric PROMs (Table 2) must be valid for children and adolescents with ACL injury. However, a paediatric-derived PROM is not currently available. Such an instrument would ensure the items covered issues that matter most to children and adolescents. The Pedi-IKDC and KOOS-Child were adapted from adult PROMs designed to assess self-reported knee function. The Pedi-IKDC has been correlated to the International Knee Documentation Committee subjective knee form—providing preliminary evidence of construct validity [10, 11]. Given that patients with a history of ACL injury may develop symptoms and signs of osteoarthritis within 10 years of the index injury [60], and the relationship between symptomatic osteoarthritis and poor quality of life [134], assessing quality of life and long-term knee function outcomes using valid PROMs may also be important.

**Table 2:** Summary of appropriate PROMs for the child with ACL Injury

Type of instrument	Scale
Health-related quality of life	Child health questionnaire [56] PedsQL [129] Pediatric PROMIS [57]
Condition- or region-specific	Pedi-IKDC [70] KOOS-Child [99]
Activity level assessment	Pediatric Functional Activity Brief Scale [39]

*IKDC* International Knee Documentation Committee, *KOOS* Knee Injury and Osteoarthritis Outcome Score

Recommendations for using PROMs in clinical practice with paediatric patients:

- Use a generic measure of health-related quality of life
- Use either the Pedi-IKDC or KOOS-Child to assess self-reported knee function
- Use the Pediatric Functional Activity Brief Scale to assess self-reported activity level

In research, it may be appropriate to include other PROMs depending on the research question. Researchers need to make decisions about the most appropriate outcome(s) when planning their study.

## Section 6: ethical considerations

This section addresses the fundamental clinical question: what are the clinician’s roles and responsibilities? Treatment decisions that involve children are among the most difficult decisions the clinician faces, especially when scientific knowledge is limited. Striking a balance between ethical principles can be especially challenging when there is a conflict of opinion. In this section, we outline the relevant ethical considerations for the clinician who treats children with ACL injuries.

It is impossible to provide specific ethical guidance that applies to all sporting injuries in adolescents and children, given the varying individual circumstances. However, it is incontrovertible that it is in the best interests of all children not to have knee and associated injuries. Therefore, injury prevention programs are fundamental to the best interests of the child. Clinicians have an obligation to support policies and practices that encourage coaches, teams/clubs and (inter)national federations to prioritise injury prevention. All parties should be committed to protecting the long-term welfare of the growing child. Nevertheless, there may be exceptional cases where parents/guardians may, with the approval of their child, rationally prioritise short-term goals. One example could be that, despite inherent risks for reinjury, an early return to sport might be a high priority for a child who has exceptional talent in a given sport.

Protecting the integrity of the knee should be the clinician’s primary focus. Decisions regarding how to protect the integrity of the child’s knee must be shared between the child, parent/guardian (surrogate decision maker), and clinician [18]. Parents have an obligation to care for their children, and bring them up to live good lives [17]. Nevertheless, parents have different perceptions of what constitutes ‘good living’ [20]. Most ethicists agree that parental influence is a positive thing [14]. However, in high performance children’s sport, parents and coaches can pressure the child and clinician to focus on short-term athletic goals at the expense of long-term welfare [54].

### Issues related to consent and obtaining consent for treatment

Children are a vulnerable population [6, 44]. In the context of treatment of ACL injury, the child is doubly vulnerable given his or her developing, but uncertain, life plans [78] and developmental stage. We can never be certain of all of the risks to normal development of the individual child [85]. It is difficult to gain legally legitimate informed consent from children in the treatment decision-making process. Therefore, the clinician needs to act as a co-fiduciary on behalf of the child, while parents give consent [88].

The clinician and/or parent(s) are obliged to serve the interests of the child above all other interests [88, 121]. This is what is meant by having a fiduciary duty to the patient. The clinician must talk with both the child and the surrogate decision makers in ways that are respectful of, and comprehensible by everyone involved [1]. In addition to avoiding conflicts of interest, the clinician must always seek the approval or assent of the child, irrespective of the parents/guardians wishes, at a communication level that matches the child’s competence [43]. The child should be present in all discussions concerning him or her, to respect his or her (emerging) autonomy [131].

### Arriving at a shared decision

There should be consensus between all parties when arriving at a decision. This consensus should be based on realistic assessments of risks and benefits and a proper consideration of the goals of the child and parent. The clinician’s responsibility is to guide this discussion with accurate information from the best quality research. There are several ethical standards that can help the clinician, child and parent(s) navigate the decision-making process, and arrive at ethically justified treatment decisions.

Some paediatric ethical standards are not identical—some aim at higher thresholds, while others accept a lower threshold of justification. There are six standards that can be helpful in different clinical scenarios in paediatric ACL injury:

1. Best interests: [72] widely used, but it is difficult to predict what is in the best long-term interests of a child.
2. Harm principle: [31] a threshold below which the clinician should not acquiesce to parent-led decision, so that the child is not harmed.
3. Parental discretion: [45, 64] parent-preference is accepted because it is not sufficiently harmful to the child for the clinician to dissent from the parent(s’) choice.
4. Costs/benefits: [29] involves risk assessment, but its application to the child means that the clinician may need to compare very different kinds of futures that may or may not eventuate.
5. Not unreasonable: [105] focuses only on the appropriateness of decisions and decision maker(s).
6. Reasonable choice: [96] a decision method that attempts to incorporate the previous five standards into a single model or intervention.

The clinician has an important role in treatment decision-making, because he or she typically has superior knowledge of treatment options, risk and benefits than children and parents. To best guide the child and his or her parent(s), the clinician must have a clear idea of the range of interventions that are (1) optimal, (2) acceptable, and (3) not desirable, and be able to justify this with reference to the best quality research and clinical experience. In many health care settings, parent(s) take responsibility for the ACL treatment decision, commensurate with the child’s assent. Where there is a lack of consensus in the decision-making process (e.g., the parent decides for something that is not recommended by the clinician), the clinician may also consider whether he or she can defend a treatment recommendation based on one of the six ethical standards.

## Section 7: future research

Management of paediatric ACL injuries is highly debated. Reflecting some of the concern and controversy is a high ratio of clinical commentaries and narrative reviews to original articles on this topic. The problem for the clinician is that there is scarce high-quality evidence that he or she can look to, to help him or her best manage paediatric ACL injuries. The scientific literature is inconsistent and limited by inferior methods that carry a high-risk of bias [35, 90]. There are no randomised trials comparing different treatment approaches or different surgical techniques. Most of the publications have only short-term follow-up; there are none with follow-up beyond 10 years. Therefore, long-term knee-health (including osteoarthritis) and quality of life is unknown.

### Methodological considerations

There are five key issues that must be addressed by future studies:

1. Most clinical studies on paediatric ACL injury are of cross-sectional or retrospective design, the study populations are often at high-risk of selection bias and include small samples. This means there is a high risk that existing research does not reflect the typical paediatric patient with an ACL injury.
2. Many studies do not provide adequate descriptions of the treatments that the patients have received, and patient adherence has not been reported. A meaningful interpretation of study outcomes is only possible with a detailed description of the surgical technique, rehabilitation, brace usage, return to sport clearance and recommendations of activity modification.
3. Many studies fail to assess the skeletal age of included participants, and few report the remaining growth of participants. Chronological age alone is an unreliable indicator of skeletal maturity. Because of this, it is difficult to know to which skeletal age group these research results apply.
4. Patients aged up to 18 years are often included in paediatric studies. This is a problem because it is likely that the patient population is a mix of skeletally mature and immature patients. Therefore, the literature may be biased toward the older patients. Having mixed populations also complicates pooling or comparing results from skeletally immature patients across studies.
5. Knowledge of pre-injury and post-treatment activity level gives important insight into a key risk factor for injury. The greater exposure a child has to potentially injurious situations (e.g., playing pivoting sport), the greater the chance of (re)injury. Activity level is a key confounding factor that is rarely accounted for in statistical analyses. This means there is a risk that estimates of secondary injury incidence may be over- or under-estimated in comparisons between studies or patient-groups.

### Research priorities

There are four research priority areas to improve prevention and outcomes of paediatric ACL injury:

1. Prospective injury surveillance studies to identify injury mechanisms and modifiable risk factors for ACL injury, combined injuries and knee reinjuries.
2. Prospective research on outcomes after surgical and non-surgical treatment (active rehabilitation alone). Long-term follow-up (beyond 10 years) is essential to answer key questions of how an ACL injury in childhood impacts physical activity, future knee-health and quality of life.
3. Research on the efficacy of different surgical techniques and characteristics (e.g., timing of surgery, graft types), and active rehabilitation programs, knee brace usage and activity modification after injury and surgery.
4. Multicentre and registry studies should be prioritised. Because of smaller numbers of ACL injuries in paediatric patients than in skeletally mature patients, specialist treatment centres, expert clinicians and researchers must prioritise collaboration.

## In memory of Dr. Allen Anderson

An excellent clinician-scientist and a keen co-worker in this project, Allen F. Anderson, MD, died in a farming accident on Sunday, November 12, 2017. This tragedy occurred shortly after he had been an active participant in this IOC consensus meeting on the topic of his life-long clinical and research passion, paediatric ACL injuries. Born on November 16, 1949, Dr. Anderson was a graduate of the University of Tennessee College of Medicine. He completed a residency in orthopaedics at Vanderbilt University and was board-certified by the American Board of Orthopaedic Surgery in general orthopaedics, with a certificate of added qualification for Sports Medicine.

Dr. Anderson was a sports medicine specialist with an interest in knee injury and ligament reconstruction, and with special interest in children’s injuries. He published more than 100 peer-reviewed journal articles and 26 book chapters, and received a patent for the invention of a paediatric ACL reconstruction system. Among numerous awards, three standouts were: being recognised as one of America’s Top Physicians 2004–2012 from Consumer’s Research Council, being elected to Best Doctors in America by his peers 2007–2008, and being Nashville Business Journal Top Doctor 2016–2017.

Dr. Anderson had many prestigious positions through his life. He served as President of the American Orthopedic Society for Sports Medicine from 2015 to 2016, and as an Associate Editor of *The Orthopedic Journal of Sports Medicine* and *The American Journal of Sports Medicine*.

Above all, he was a true friend and colleague whom you could go to with problems and challenges, not the least among our youngest patients. Allen will be greatly missed by us all.

## Electronic supplementary material

Below is the link to the electronic supplementary material.

Supplementary material 1 (DOCX 65 KB)

Supplementary material 2 (DOCX 20 KB)
