# Supplementary material for: 2018 International Olympic Committee consensus statement on prevention, diagnosis and management of paediatric anterior cruciate ligament (ACL) injuries
Source: Knee Surg Sports Traumatol Arthrosc. 2018 Feb 17;26(4):989–1010. doi: 10.1007/s00167-018-4865-y (PMC5876259; doi:10.1007/s00167-018-4865-y)
Supplement: Supplementary file 1 — Supplementary material 1 (DOCX 65 KB) [file 167_2018_4865_MOESM1_ESM.docx]

# Delphi consensus process statements

1. Diagnostic tests and imaging
2. Methods for skeletal age assessment
3. Surgical techniques (transphyseal vs. physeal-sparing)
4. Indications for surgical treatment
5. Risks associated with surgical treatment (e.g. growth disturbance, joint angulation)
6. Uncertainties and limitations regarding the paediatric ACL graft
7. Rationale for non-surgical treatment
8. Disadvantages/risks associated with non-surgical treatment (e.g. secondary meniscal injury)
9. Management of associated injuries (e.g. meniscus, articular cartilage)
10. Rehabilitation guidelines
11. Functional tests for treatment decision making and clearance to return to unrestricted activity
12. ACL injury prevention
13. Managing re-injury risk
14. Paediatric patient-reported outcomes
15. Guidelines for long-term follow-up
16. Development of posttraumatic osteoarthritis
17. Influence of treatment approach on development of posttraumatic osteoarthritis
18. Development of a paediatric ACL treatment outcome registry
