# Supplementary material for: 2018 International Olympic Committee consensus statement on prevention, diagnosis and management of paediatric anterior cruciate ligament (ACL) injuries
Source: Knee Surg Sports Traumatol Arthrosc. 2018 Feb 17;26(4):989–1010. doi: 10.1007/s00167-018-4865-y (PMC5876259; doi:10.1007/s00167-018-4865-y)
Supplement: Supplementary file 2 — Supplementary material 2 (DOCX 20 KB) [file 167_2018_4865_MOESM2_ESM.docx]

# Exercise examples for each phase of paediatric ACL rehabilitation

|  |  |
| --- | --- |
| Phase 1 | - Stationary bike - Active extension (unloaded) - Quads setting - Squat variants with and without support - Single limb standing (control of isometric terminal knee extension) - Closed chain hip and pelvis control exercises |
| Phase 2 | - Single limb standing control of dynamic terminal knee extension - Single leg squats - Bridging - Squats on BOSU - Step-ups (front and lateral) - Lunge onto BOSU |
| Phase 3 | - Bulgarian split squats (progress by adding hand weights – dumbbells or kettlebells) - Stair jumps (double and single leg) - Split squat jumps on BOSUs - Hopping and landing emphasising shock absorption and avoiding dynamic knee valgus - Lateral, frontal and backwards agility exercises - Running direction change exercises (progress from wide turn to tight turn/tight cut, from around a stationary object to an opponent) - Leg press - Quads strength with leg extension machine |
| Phase 4 | Injury prevention (refer to Section 1 of the consensus statement, and FIFA 11+ for Kids manual^1^ for guidance) |

References

**1.** Rössler R, Faude O, Bizzini M, Junge A, Dvorak J. *FIFA 11+ for Kids manual: a warm-up programme for preventing injuries in children's football*: FIFA Medical and Research Centre (F-MARC).
